# The Role of Sounds and Music in Emotion and Cognition

**DOI:** 10.3390/brainsci14030192

**Published:** 2024-02-21

**Authors:** Laura Piccardi, Massimiliano Palmiero, Raffaella Nori

**Affiliations:** 1 Department of Psychology, Sapienza University of Rome, 00185 Rome, Italy; 2 San Raffaele Cassino Hospital, 03043 Cassino, Italy; 3 Department of Communication Sciences, University of Teramo, 64100 Teramo, Italy; mpalmiero@unite.it; 4 Department of Psychology, University of Bologna, 40127 Bologna, Italy; raffaella.nori@unibo.it

It is widely agreed upon that both natural and man-made sounds, including music, profoundly impact our emotions and cognitive abilities, such as our attention, memory, problem-solving, decision-making, and creativity. Numerous studies have proven that the impact of auditory stimuli on our emotions and cognition is influenced by various factors, including the characteristics of the stimuli, the nature of the tasks being performed, and individual differences in processing sounds and music.

Using a meta-analytical approach, Roman-Caballero et al. [1] explored the causal impacts of learning to play a musical instrument on cognitive abilities and academic success during the schooling years. While they discovered an initial culturally and economically favourable background for individuals who chose to learn an instrument or pursue music studies, they also witnessed an impact from such practices. It is undeniable that engaging in the intricate process of learning to play a musical instrument for an extended period can result in neurocognitive adaptations, leading to significant improvements in overall cognitive abilities and academic performance. These authors found that learning to play an instrument during school has a small but important effect on a person’s cognitive abilities and academic performance. 

To provide evidence for the significance of musical instrument practice, multiple studies have shown that musicians excel in various cognitive tasks compared with non-musicians (see [2]). Specifically, musical training is believed to enhance various cognitive and emotional abilities, including verbal memory, fluency, perception, creativity, spatial skills, IQ scores, and empathy [3,4,5,6,7,8,9,10,11,12]. However, after evaluating evidence produced by other researchers, Schellenberg and Lima [13] concluded that causal inferences cannot be made. Specifically, these authors suggested that there is no conclusive evidence to support the claim that music training has far-reaching cognitive benefits that can be generalised to other domains, which is consistent with the findings obtained from other types of training. Nevertheless, Schellenberg and Lima [13] advocated for music to be included in school curricula and funded research due to its intrinsic value.

The debate about music’s impact revolves around its effects on cognitive development and abilities. Yet, there is also research leveraging music to enhance the quality of life, mood, and cognitive functions of patients with brain damage or neurodegenerative diseases.

This Special Issue consists of five Articles and one Review. Three of the studies focus on the performance of cognitive tasks by musicians and non-musicians, particularly in working memory, creative thinking, and vocal processing. Specifically, Pino et al. examined the impact of formal musical education on the connection between working memory and divergent thinking among musicians. Their findings indicated that years of formal musical training influenced the link between working memory and divergent thinking, implying that music amplifies the beneficial impact of advanced cognitive processes on the capacity for divergent thinking.

Nussbaum et al. found that musicians are better than non-musicians at recognising vocal emotions. They compared event-related potentials to acoustically manipulate voices between the two groups to understand the difference at the brain level. The results suggest that musical ability impacts how people use sound cues to evaluate emotions later. In a pilot study, Ramirez-Melendez and Reija observed that the emotional brain is active in professional drummers while playing creative music at various levels. They completed three tasks while their brain activity was recorded: repetitive drum playing, pattern-based improvisation, and free improvisation. These actions require large amounts of attention. The study found higher levels of positive emotions during both pattern-based and free improvisation compared with the baseline and higher positive emotions during free improvisation compared with pattern-based improvisation. These results suggest that feeling positive is linked to coming up with new ideas while playing the drums and that the more freedom there is in the creative process, the greater the positive impact. Although the study provides preliminary results, their significance could have an impact on the realms of music-based therapy interventions and music education. 

The remaining contributions specifically explored how music can influence mood and, in turn, enhance performance. These contributions were tailored towards engaging non-experts rather than experts. In particular, one study utilised music in a therapeutic setting. 

Palmiero et al. performed a study in which they manipulated participants’ moods by allowing them to select their own happy, sad, or neutral music to listen to for ten minutes. Subsequently, the participants were asked to create two artistic drawings. Three impartial judges assessed the drawings from the negative mood group as more creative and emotional than those from the other two groups. These findings validate that sad music can positively impact artistic creativity, potentially due to an increased inclination for mood regulation or, more likely, as a consequence of experiencing a mixed emotional state.

Hamiduzzaman et al. looked at how person-centred, culturally appropriate music affects the psychological well-being of residents with advanced dementia. The study was conducted in five rural, residential aged care homes. The authors employed the Music in Dementia Assessment Scale (MiDAS) and conducted interviews and focus groups with aged care staff and musicians to explore the residents’ interest, response, initiation, involvement, enjoyment, and general reactions. According to the findings, incorporating music into care plans could reduce agitation and enhance emotional well-being among residents in rural aged care facilities.

The Review, on the other hand, placed its focus on the neurological and psychological consequences of music while also presenting the remarkable clinical importance of music-based therapies. Toader et al., in their insightful Review, explained how music engages several brain areas and intricate neural connections, thus highlighting the complexity of this fascinating phenomenon. Music-based therapies are effective in rehabilitating people with depression, anxiety, and neurological disorders such as stroke. The Review’s notable contribution lies in its emphasis on the reciprocal interactions between music and the limbic system, providing a clear understanding of how music influences our emotional states through neurobiological mechanisms. The Review focuses on the neural networks that allow for synchronisation between external rhythmic stimuli and internal neural oscillators in motor systems and coordination. These findings are important for developing music-based rehabilitation methods; adding rhythmic elements could improve current therapeutic approaches.

Finally, in light of these studies, we would like to emphasise the significance of the authors’ contributions in showcasing the positive effects of music on mood regulation and cognitive performance. Whether it be through playing an instrument or simply listening to music, these benefits apply to individuals of all expertise levels and mental health statuses.
